# Hydrogels in Veterinary Vaccine Development: Types, Mechanisms, and Applications

**DOI:** 10.3390/gels11060468

**Published:** 2025-06-18

**Authors:** Peisen Zhao, Yuwei Yang, Lingxue Yu, Guoxin Li, Dandan Zhu

**Affiliations:** 1 Shanghai Veterinary Research Institute, Chinese Academy of Agricultural Sciences, Shanghai 200241, China; 2 School of Chemistry and Chemical Engineering, Shanghai Jiao Tong University, Shanghai 200240, China

**Keywords:** hydrogel, vaccine delivery, immunomodulation, biocompatibility

## Abstract

This review examines the potential and challenges of using hydrogel vaccine delivery systems in animal immunization. Traditional methods face issues like low immunogenicity, reliance on cold chains, and inefficient delivery, limiting their use in modern animal husbandry. Hydrogels offer a promising solution due to their biocompatibility, controlled drug release, and immune regulation. This paper highlights hydrogels’ benefits, such as mimicking natural infection through sustained antigen release, boosting antigen-presenting cell activity, activating immune responses, and forming barriers at mucosal sites to prevent pathogen invasion. Additionally, innovative delivery methods like microneedle patches and nasal sprays show promise in enhancing convenience and compliance in animal vaccination. By combining interdisciplinary efforts and technological advancements, the hydrogel vaccine delivery system is anticipated to be crucial in preventing animal diseases, supporting sustainable animal husbandry, and ensuring global animal health and food safety.

## 1. Background

Vaccines play an irreplaceable core role in animal disease prevention and control systems [1]. The infection rate and transmission risk of the target disease can be significantly reduced through vaccination, thereby building an immune barrier in the animal population. This active prevention strategy not only effectively controls the mortality of livestock and poultry but also provides the basic guarantee for the sustainable development of animal husbandry by blocking the spread of pathogens in the breeding environment and, at the same time, indirectly protects humans from some zoonotic diseases [2,3]. However, with the development of the breeding industry and the increasing focus on animal health, its limitations have become increasingly evident. In veterinary practice, traditional animal vaccines are painful. Additionally, some traditional vaccines have weak immunogenicity, which makes it difficult to stimulate an immune response of sufficient strength and duration in animals, resulting in poor immunity [1]. In addition, traditional vaccines have poor stability and require strict cold chain conditions during storage and transportation. Otherwise, they can easily become inactivate, which has resulted in great inconvenience for the use of vaccines in some areas with imperfect cold chain facilities [4,5]. Furthermore, some vaccines have severe side effects, which may cause adverse reactions in animals and affect their health and production performance [6]. These problems not only increase the difficulty of the work of veterinarians but also pose challenges to animal health and the sustainable development of the breeding industry.

At the same time, in large-scale farming scenarios, the delivery efficiency of traditional vaccines is low. For example, in intensive farming, many animals need to be vaccinated. The traditional one-by-one injection methods not only consume considerable manpower, material resources, and time but also may cause stress to the animals, affecting their growth and health [7]. Therefore, more efficient, safer, and stable animal vaccines and delivery systems are urgently needed.

The abovementioned limitations of traditional animal vaccines have severely constrained the effectiveness of animal disease prevention and control and the sustainable development of animal husbandry. In this context, scientists are committed to identifying novel vaccine delivery technologies that can overcome these bottlenecks. In recent years, the rapid development of hydrogel technology has led to new discoveries. As novel vaccine delivery vehicles, hydrogels, owing to their unique physicochemical properties and biocompatibility, can effectively solve many problems faced by traditional animal vaccines, leading to a potential revolution in the field of animal immunization [8]. As an important achievement in the field of materials science, hydrogel technology has brought new opportunities and solutions to the vaccine field. As a highly water-absorbent polymer network, hydrogels exhibit unique biocompatibility, biodegradability, and controlled drug release characteristics [9]. Their unique physicochemical properties endow them with enormous potential for application in the biomedical field, especially in the delivery of drugs and vaccines [10]. On the one hand, hydrogels can be used as vaccine carriers to achieve slow release of the vaccine and prolong the action time of the antigen in the body, thereby enhancing the immune effect [11]. On the other hand, hydrogels can be modified and functionalized to achieve targeted delivery to specific tissues or cells, improving the delivery efficiency and specificity of vaccines [12]. In addition, hydrogels have good biocompatibility, which can reduce the stimulation and adverse reactions of vaccines in the animal body. These characteristics of hydrogel technology make it possible to address the pain associated with traditional animal vaccines and are expected to promote technological innovation in the field of animal vaccines.

This review aims to discuss the latest research progress on hydrogels in the field of vaccine delivery; the types, characteristics, loading, and release mechanisms of hydrogels; and the role of hydrogels as adjuvants. Furthermore, the prospects of hydrogels as vaccine delivery systems in veterinary application scenarios are also discussed to provide new ideas for researchers and developers in the field of veterinary vaccine R&D.

## 2. Hydrogel

### 2.1. Types and Applications of Hydrogels

Hydrogel is a soft material with a three-dimensional cross-linked network structure that can absorb and retain large amounts of water in its structure. According to their source, hydrogels can be divided into two types: natural and synthetic. Natural hydrogels are prepared from natural product polysaccharides or biological-source proteins and have good biocompatibility and biodegradability, but there may be allergen issues [13]. Synthetic hydrogels have more advanced performance potential, such as high water absorption capacity and improved physical and chemical stability, but their biological activity and compatibility may be challenging [14]. Because of the special physicochemical properties of hydrogels, they are very suitable as vaccine delivery systems: their mechanical strength can protect vaccine components from external environmental damage, their biocompatibility can reduce side effects in the immune response, and their responsive function can be stimulated (Table 1). Precise control of vaccine release can be achieved, thereby improving the immune effect of vaccines [15,16,17].

### 2.2. Loading of the Hydrogel

Hydrogels have been extensively studied as delivery vehicles. Their unique 3D network structure and high water content enable them to adsorb and release drugs effectively [23]. The hydrogel is loaded mainly through physical interactions and chemical bonds.

Physical adsorption is a simple and effective way to load drugs on hydrogels. Drug molecules are loaded into the 3D network structure of a hydrogel through noncovalent physical interactions (such as van der Waals forces, hydrogen bonding, π–π stacking, and electrostatic forces) [24]. The advantages of physical adsorption are simple operation, mild conditions, and suitability for the loading of heat-sensitive drugs [25,26]. In drug delivery systems, hydrogels can be used not only for the delivery of small-molecule drugs but also for the delivery of macromolecular drugs such as protein and nucleic acid drugs. Through physical adsorption, the hydrogel can effectively protect these macromolecular drugs from degradation by enzymes in the body and achieve sustained release at the target site [27]. For example, owing to their excellent biocompatibility and drug release characteristics, nanocellulose-based hydrogels have been extensively investigated for the delivery of various drugs [28]. Like drugs loaded in hydrogels, vaccines can also be loaded in hydrogels through physical interactions, that is, weak forces. The reported loading methods are based on electrostatic interactions [29], H bond self-assembly [30], etc.

Chemical bonding is the immobilization of vaccine components into a hydrogel network through covalent bonds [31]. This method can improve the stability and controlled-release performance of vaccines. For example, the metal-phenolic network (MPN) hydrogel vaccine platform can effectively stimulate the body’s humoral immune response by covalently immobilizing the vaccine components in the hydrogel network [32]. This hydrogel vaccine could significantly enhance the neutralizing antibody response to the rabies virus after a single immunization, with the antibody levels increasing by 4.3-fold and 1.8-fold compared with those of the traditional inactivated rabies vaccine and aluminum adjuvant, respectively [32]. The advantage of chemical conjugation is that it can provide a more stable vaccine delivery system and reduce the early release and degradation of vaccines in the body [33]. Chemical bonding is stronger than physical adsorption but requires a certain activation energy. In addition, hydrogels can also be composited with nanoparticles. Vaccine components can be encapsulated in nanoparticles, and a composite hydrogel system with specific functions can be formed by combining the nanoparticles with hydrogels [22]. Nanoparticles can improve the mechanical properties and delivery ability of hydrogels as well as achieve targeted delivery and controlled release [34]. In the delivery of some vaccines that require precise targeting of immune cells, nanoparticle composite hydrogels have significant advantages. For example, nanomaterials such as graphene oxide and low-molecular-weight polyethyleneimine can be combined with hydrogels to form a composite system with good biocompatibility and biodegradability for the delivery of mRNA vaccines [21]. The application of this composite material in the delivery of mRNA vaccines has demonstrated its potential in cancer immunotherapy, significantly increasing the number of antigen-specific T cells and inhibiting tumor growth. In addition to serving as vaccine carriers, hydrogels can also function as enzyme-insulating matrices. For instance, a chiral peptide-based hydrogel (CP-CNDS) developed by He et al. was shown to effectively entrap digestive enzymes such as trypsin, chymotrypsin, and lipase within its three-dimensional network. This entrapment substantially suppressed enzymatic activity, thereby mitigating potential tissue damage in enzyme-rich environments. Such findings underscore the utility of hydrogels as protective biological barriers, particularly in mucosal vaccine delivery systems where enzymatic degradation poses a significant obstacle to antigen stability and bioavailability [35].

### 2.3. The Physicochemical Properties of Hydrogels

The physical and chemical properties of hydrogels play a crucial role in the design and application of vaccines as carriers.

The three-dimensional structure and high water absorbency of hydrogels enable them to effectively protect and release antigens. By adjusting the crosslinking density, swelling behavior, and response to external stimuli such as pH, temperature, or ionic strength, the release profile of encapsulated bioactive compounds can be precisely controlled, thereby maximizing therapeutic effects and minimizing side effects [36].

The surface properties of hydrogels, such as hydrophilicity and hydrophobicity, also affect their interaction with immune cells. Research indicates that increasing the surface hydrophobicity of particles significantly enhances antigen internalization efficiency within dendritic cells, thereby boosting immune responses [37]. The pore structure of hydrogels is crucial for the migration, invasion, and regulation of immune phenotypes of immune cells. Hydrogels with porous structures not only facilitate nutrient transport and metabolic waste removal but also provide more space for cell function [38].

In vaccine delivery systems, the injectability and self-healing ability of hydrogels make them an ideal biomaterial platform. By designing shear-thinning and self-healing polymer-nanoparticle hydrogels, local immune cell recruitment and programming can be achieved, enabling in vivo immune modulation [39]. The dynamic covalent cross-linking properties of hydrogels allow them to serve as injectable vaccine adjuvants, providing sustained antigen release and significantly enhanced humoral immunity [40]. Additionally, the physical characteristics of hydrogels, such as size, structure, shape, mechanical strength, etc., also influence their phagocytic regulation by immune cells, biological distribution, and targeting capabilities. These properties can be precisely engineered to elicit desired innate immune responses, playing a role in cancer immunotherapy [41].

## 3. Strategies for Priming the Immune Response with Hydrogel-Based Vaccines

The mechanism of the immune response to vaccines is a complex process that involves the coordination of various immune mechanisms. Vaccines provide protection by stimulating the body’s immune system to induce an immune response against specific pathogens. First, after vaccination, the body’s innate immune system responds quickly by recognizing the antigens in the vaccine and initiating an inflammatory response. This stage mainly involves the participation of innate immune cells such as dendritic cells, macrophages, and neutrophils [42].

Next, the adaptive immune system is activated, and B cells and T cells are activated. B cells differentiate into plasma cells under the stimulation of antigens, which produce specific antibodies. These antibodies can neutralize pathogens or mark pathogens for recognition and clearance by other immune cells [43,44]. Moreover, T cells recognize antigen peptide–MHC complexes on the surface of antigen-presenting cells to exert cytotoxicity or function as helper B cells and other immune cells [45].

The immune response to a vaccine is not limited to a single immunization route but is achieved through the synergetic action of various mechanisms. Vaccines can simultaneously activate humoral immunity and cellular immunity by combining different adjuvants, thereby increasing the strength and durability of the overall immune response [46,47].

The mechanisms of action of adjuvants can be roughly divided into two types: delivery vehicles and immunostimulatory agents [48]. As delivery vehicles, adjuvants can act as depots to help deliver antigens to draining lymph nodes, promote antigen uptake by antigen-presenting cells (APCs), and protect antigens from harsh environments. Adjuvants with immunostimulatory activity may promote the recruitment and activation of APCs and T cells, enhance the function of APCs, and guide T-cell differentiation and immunoglobulin isotype switching [49].

**Figure 1:**
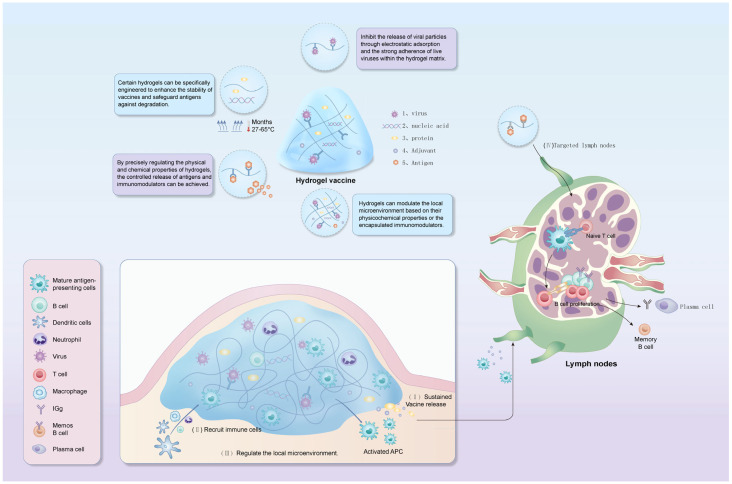
The diagram illustrates the mechanism by which hydrogel-encapsulated antigens and immune modulators enhance immune protection efficiency.

Similarly, as adjuvants, hydrogels can enhance the effects of vaccines by prolonging antigen release, enhancing phagocytosis and antigen presentation by phagocytes, promoting local inflammation and regulating the immunosuppressive microenvironment, as illustrated in Figure 1 [40,50,51].

### 3.1. Prolonged Antigen Release

The high water content and biocompatibility of hydrogels make them ideal carriers of antigens and immunomodulators. By regulating the physical and chemical properties of a hydrogel, the controlled release of antigens and immune modulators can be achieved, resulting in persistent immune stimulation in vivo [52,53]. For example, dynamic covalent hydrogels (DCHs) are used as single-dose vaccine adjuvants and can continuously release recombinant protein antigens for 10 to 30 days, significantly improving vaccine efficacy. This sustained release mechanism not only improved the antibody titer but also demonstrated its broad applicability in different antigen tests [40].

This slow-release process simulates the persistent state of pathogens during natural infection in the body. During the natural infection process, pathogens are not recognized by the immune system all at once, but as they proliferate and spread throughout the body, they continuously expose antigens to the immune system. Through this simulation, the hydrogel vaccine can prolong the contact time between the antigen and antigen-presenting cells (APCs). APCs, such as dendritic cells, need a certain amount of time to recognize, uptake, and process antigens before passing antigen information to other immune cells [50,54]. Therefore, prolonged contact time enables APCs to more fully complete these processes, thereby more effectively activating the immune system [55].

Recent preclinical investigations have further demonstrated the immunological advantages of hydrogel-based vaccines in animal models. In one study, a PLGA-PEG-PLGA hydrogel encapsulating a Newcastle disease virus DNA vaccine enabled immunized poultry to maintain protective antibody titers for more than eight weeks, in contrast to only four weeks observed in the conventional vaccine group [56]. Similarly, a chitosan–hyaluronic acid composite hydrogel used to deliver a Toxoplasma gondii antigen elicited a 2.7-fold elevation in total IgG levels and a marked increase in IgG1 production, indicating a robust and sustained Th2-type humoral response [57]. Furthermore, hydrogel formulations incorporating plant virus-based subunit antigens were capable of inducing durable antibody responses in mice, with immunity persisting for over 20 weeks—significantly longer than that achieved by soluble antigen counterparts [58]. These findings collectively highlight the capacity of hydrogel-based delivery systems to prolong antigen exposure, potentiate humoral immune responses, and extend the duration of protective immunity, thereby offering considerable promise for veterinary vaccination programs.

### 3.2. Enhancement of Phagocytosis and Antigen Presentation by Phagocytes

The 3D network structure of the hydrogel allows the infiltration and migration of immune cells, forming an “immune hotspot” at the injection site that is conducive to the immune response [59]. For example, one study developed a hyaluronic acid-based biomimetic nanocomposite hydrogel system that can recruit host immune cells in vivo to elicit a powerful and durable humoral immune response [60]. By combining with N-trimethylchitosan nanoparticles (TMC/NPs), this hydrogel system can not only prolong the release time of vaccine antigens but also significantly enhance the maturation of dendritic cells, thereby improving the immune response. Immune adjuvants encapsulated in hydrogels, such as polyinosine-polycytidylic acid (poly I:C) (TLR3 agonist), monophosphoryl lipids (TLR4 agonist), and CpG oligodeoxynucleotide (ODN) (TLR9 agonist), can also further enhance the immune response by recruiting and activating immune cells. Take CpG as an example. It is a DNA fragment containing unmethylated CpG dinucleotides that can be recognized by TLR9 on the surface of immune cells and can effectively induce the activation of immune cells and the secretion of cytokines, thereby enhancing the immunity of the body [61,62]. When a hydrogel is loaded with CpG and delivered around immune cells, CpG can be taken up by immune cells, activate the TLR9 signaling pathway, and promote the activation and proliferation of immune cells and the secretion of cytokines.

Additionally, another study discussed the use of tetrapeptide hydrogels as potential adjuvants for H7N9 vaccines. Studies have shown that tetrapeptide hydrogels can significantly improve the protective effect of vaccines and increase the microneutralization antibody titer and hemagglutination inhibition titer in mice after infection with the H7N9 virus [63]. These results indicate that hydrogels can not only act as vaccine carriers but also enhance the immune response through their physical properties and biocompatibility.

The adjuvant effect of the hydrogel itself and the synergy of loaded immune regulatory molecules can achieve better immune effects with a reduced dose of exogenous adjuvant, thereby reducing the risk of systemic toxicity and improving the safety and tolerance of vaccines [40].

### 3.3. Regulation of the Local Inflammatory Response

The porosity of the hydrogel has a significant regulatory effect on the polarization (M1/M2 balance) of macrophages. The greater porosity allows more immune molecules (such as anti-inflammatory factors such as IL-4 and IL-10) and nutrients to infiltrate the hydrogel, promoting the survival and function of M2 macrophages. For example, studies have shown that hydrogels with larger pore structures can effectively promote the transformation of macrophages into the anti-inflammatory M2 phenotype, thereby facilitating tissue regeneration and healing [38]. M2 macrophages suppress excessive inflammation and accelerate tissue repair by secreting anti-inflammatory factors (such as TGF-β) and prorepair factors (such as VEGF). A notable example is the work by Shi et al., who developed a naturally derived hydrogel system composed of keratin, protocatechuic aldehyde, and Fe^3+^ ions. This dual dynamic crosslinked hydrogel exhibited excellent injectability, self-healing, and tissue adhesion properties. More importantly, it significantly promoted M1-to-M2 macrophage polarization in diabetic wound models and enhanced re-epithelialization and collagen deposition [64]. On the other hand, the lower porosity may indirectly maintain the proinflammatory activity of M1 macrophages by limiting the migration and signal transmission of immune cells, thereby enhancing pathogen removal ability in the early stage of infection. Some studies have shown that the surface characteristics of a material, such as roughness and hydrophilicity, can significantly affect the polarization state and function of macrophages [65].

The porosity of hydrogels significantly regulates macrophage polarization (the M1/M2 balance). Larger porosity allows more immune molecules, such as anti-inflammatory factors like IL-4 and IL-10, and nutrients to penetrate into the hydrogel, promoting the survival and function of M2-type macrophages. For example, studies have shown that hydrogels with larger pore structures effectively promote the conversion of macrophages to the anti-inflammatory M2 phenotype, thereby aiding tissue regeneration and healing [66]. Self-healing hydrogels can also activate anti-inflammatory signaling pathways (such as the Nrf2/ARE pathway) through the release of metal ions (such as magnesium ions) to further regulate the macrophage phenotype. Moreover, cell proliferation and differentiation can also be promoted through other signaling pathways, such as the PI3K/Akt pathway, to further support tissue regeneration [67,68]. Additionally, hydrogels and extracellular vesicles (EVs) can also regulate vaccine efficacy and local inflammation by carrying immune-regulatory microRNAs (miRNAs). For example, miRNAs such as miR-192 and miR-21 can improve vaccine efficacy and regulate the local inflammatory response in aged mice [69].

### 3.4. Precise Release in Time and Space

The intelligent design of a hydrogel vaccine delivery system can achieve accurate spatiotemporal delivery of antigens and adjuvants, thereby improving the immune effect of vaccines.

A common strategy is to utilize stimuli-responsive hydrogels. Temperature-sensitive hydrogels can form a gel at the injection site according to changes in body temperature to achieve slow release of the vaccine. For example, in the delivery of an influenza vaccine, a heat-sensitive hydrogel rapidly gelled at body temperature prolonged the duration of the vaccine in the nasal mucosa and enhanced the immune effect [11]. In addition, the heat-sensitive hydrogel not only prolonged the residence time of the vaccine but also improved the immune effect by enhancing the transepithelial transport of antigens. Studies have shown that heat-sensitive hydrogels can enhance the systemic immune response and mucosal IgA immunity by degrading the ZO-1 protein in nasal epithelial tissue and promoting antigen transport through the intercellular pathway [11]. In addition to temperature sensitivity, magnetic and pH-responsive hydrogel systems have been developed for more sophisticated navigation and delivery. Zhong et al. constructed an ultra-soft magnetic hydrogel robot that demonstrated dual-modal locomotion and dynamic deformation in physiological environments, offering new possibilities for targeted vaccine transport through tissue barriers [70].

Another strategy is to modify the surface of the hydrogel to target it. Specific ligands, such as antibodies and peptides, are linked to the surface of the hydrogel. These ligands can specifically bind to receptors on the surface of target cells to achieve targeted delivery to specific cells. For example, researchers have successfully achieved the selective capture of endothelial progenitor cells (EPCs) by immobilizing a CD34 antibody on hyaluronic acid (HA) hydrogels. This antibody-modified hydrogel surface could significantly improve the adhesion ability of EPCs while having no significant effect on the adhesion of macrophages [71]. Furthermore, in another study, the covalent immobilization of proteins or peptides was successfully achieved by the introduction of SpyTag and SpyCatcher protein domains into polyethylene glycol (PEG) hydrogels. This method is not only simple and gentle but also enables biochemical modifications of the hydrogels, thereby controlling the interaction between cells and materials. This cell-friendly site-specific ligation strategy has great potential in driving specific cellular outcomes [72]. NPs are used to encapsulate ligands that can bind to DC surface receptors (e.g., TLRs, TNF-receptors (TNF-Rs), etc.) and can impart the ability to target DCs [73]. At the same time, DC-targeting nanoparticles can also be loaded into hydrogels to target them.

## 4. Technological Innovations and Prospects in the Context of Veterinary Vaccines

### 4.1. Technological Innovation of Cold Chain Substitution

Hydrogels have a certain cold chain replacement potential. Some hydrogels can be specially designed to improve the stability of vaccines and reduce their dependence on cold chains. MarcoDufort developed a reversible PEG-based hydrogel platform formed through dynamic covalent boronate ester cross-linking for the encapsulation, stability, and on-demand release of biologicals. This hydrogel is capable of stabilizing a variety of biological agents, including protein vaccines and whole viruses, at temperatures up to 65 °C [74]. In another study, Sim designed a heparin-based temperature-sensitive injectable hydrogel system for the sustained release of proteins. This system was able to form hydrogels under physiological conditions and exhibited good biocompatibility and protein release characteristics in vivo [75]. Native starch from Plectranthus esculentus tubers was carboxymethylated and acetylated, and the hydrogel derivatives formed were used to stabilize the Newcastle disease (ND) LaSota vaccine. These modified starches not only maintained vaccine stability at low temperatures (5 ± 2 °C) but also exhibited excellent stability at high temperatures (37 ± 1 °C). Under conditions of simulated cold chain failure, these modified starch stabilizers caused only a slight decrease in the vaccine titer, which was far superior to that of traditional peptone stabilizers [76].

Modification of hydrogels, such as the addition of protective agents and stabilizers, can further improve the stability of vaccines under non-cold chain conditions. The development of hydrogel vaccine delivery systems that can be stored and transported at room temperature will greatly reduce the storage and transportation costs of vaccines.

### 4.2. Solutions for Intensive Farming

Under intensive breeding systems, the prevention and control of animal diseases are facing the dual pressures of efficiency bottlenecks and biosafety risks. The intensive farming model has significantly improved production efficiency through high-density feeding, but at the same time, it has also reconstructed the ecological pattern of animal disease transmission. In a closed aquaculture environment, space compression of animal populations leads to fundamental changes in pathogen transmission dynamics [77]. Advancements in hydrogel technology could offer diverse solutions in this field.

As adjuvants, hydrogels have good functionality and can enhance the immunogenicity of vaccines and prolong the protective period of vaccines. For example, a DNA-based immunostimulatory hydrogel was used to increase the protective efficacy of nanotoxin vaccines. Through the incorporation of a DNA hydrogel containing embedded immunostimulatory CpG motifs, this system markedly enhanced the efficiency of antibody production and antigen-specific cellular immunity, as evidenced by the observed immune response [38]. Modaresifar K et al. developed an injectable hydrogel-based system containing biodegradable nanoparticles [78]. This system was designed to load human basic fibroblast growth factor (bFGF) nanoparticles, which were subsequently introduced into hydrogels. This system converts the cargo to a gel and releases it at a physiological temperature. This delivery system has been shown to generate a strong immune response in the presence of antigen molecules. After injection, gelation of a temperature-sensitive hydrogel (such as poloxamer 407/188) is triggered by body temperature to form a 3D network structure, and the drug release period is prolonged for more than 70 days [20]. In another study, a poloxamer 407/188 hydrogel was used in a vaccine delivery system, and the results showed that it could induce a strong and durable immune response in the body, thus improving the immunogenicity of vaccines [79].

As novel adjuvants, hydrogels can transform the “pulse” protection of vaccines from a single immunization to “platform” protection that continuously covers the production cycle, especially in the field of animal husbandry, which can effectively cope with the growth cycle and immune lapse of animals to provide a full-cycle protection scheme for large-scale farming (Table 2).

Hydrogel vaccines form a protective barrier in the respiratory and digestive tract mucosa. These hydrogels can mimic the properties of natural mucus to provide a dynamic defense barrier against the invasion of viruses and other pathogens. For example, studies have shown that lignin-based mucus-mimetic antiviral hydrogels can effectively inhibit viral infection through their tunable porosity and enzyme stability [80]. Mucus-inspired self-healing hydrogels also have the potential to prevent viral infection, and these hydrogels protect cells by trapping viral particles [81]. This barrier not only physically blocks the virus but also enhances the local immune response. In terms of vaccine delivery, cationic cholesteryl pullulan nanogels, as effective nasal vaccine delivery systems, are able to induce a protective immune response in the respiratory mucosa [82]. This delivery system takes advantage of the unique immune characteristics of the respiratory mucosa to promote the generation of antigen-specific humoral and cellular immune responses. Oral biomimetic viral vaccine hydrogels enhance the adhesion of mucosal surfaces through their virus-like structure, thereby improving the uptake efficiency of antigen-presenting cells [83]. The self-healing hydrogel nasal spray vaccine developed by the team of Academician Chen Chunying at the National Center for Nanoscience and Technology delivers antigens through the intranasal route to stimulate a protective immune response against respiratory viruses. This hydrogel vaccine forms a stable “mucosal mask” in the nasal cavity, which significantly increases the stability and residence time of antigens on mucosal surfaces, thereby enhancing the antigen-specific immune response [84].

Hydrogel vaccine delivery systems exhibit significant species-specific advantages when applied to major economic animals thanks to recent breakthroughs in technological innovation.

In poultry farming, hydrogel technology can enable the microencapsulation delivery of coccidia vaccines. Carrageenan and alginate gels can serve as carriers for encasing Eimeria oocysts, distributing them through automated nozzles into microcapsules with diameters of 2.3–2.5 mm, which are then uniformly suspended in the gel. This process effectively protects the oocysts and allows their release when needed, thereby enhancing vaccine stability and efficacy [85]. Moreover, the combination of agarose and alginate has demonstrated superior performance in other biological applications. In one study, researchers prepared monodisperse microcapsules using external gelation techniques, which exhibited excellent thermal stability and protective effects, effectively releasing encapsulated cells under different pH conditions. The successful application of this technology provides strong support for the potential of biomaterials in vaccine delivery and other biomedical applications [86].

In pigs, the vast majority of pathogenic microorganisms can invade the body through mucosal channels (respiratory tract, digestive tract, reproductive tract, etc.), causing illness [87]. Mucosal immunity is becoming increasingly extensive and efficient in the application of vaccinations [88]. Experiments have shown that, in terms of the protective effect of PEDV, oral immunization > nasal spray immunization > intramuscular injection [89]. The greatest problem facing oral vaccines is the “passing the stomach effect”, and hydrogels may be a good solution to this phenomenon. For example, the oral administration of PEDV dissolved in Alg-CS gel was able to induce a high-level and durable mucosal immune response in mice. Compared with those in the oral administration of PEDV alone, the IgA and IgG levels in the Alg-CS+PEDV gel group at 12 d and 24 d after immunization were greater. Oral administration combined with subcutaneous immunization significantly increased the levels of IgG and IgA, showing an enhanced immune effect [90]. Alginate-chitosan-coated layered double hydroxide nanocomposites can protect antigens in the stomach and perform controlled release in the intestinal tract [91]. Chitosan-based hydrogels also exhibit strong mucoadhesion and prolonged antigen retention in the intestinal wall, thereby promoting immune activation [92]. Researchers have compared the replication characteristics of different attenuated PRRSV strains in the nasal mucosa. The results revealed that the replication efficiency of some strains in the nasal mucosa was significantly greater than that of others, which further supported the effectiveness of nasal spray immunization [93]. Moreover, mucosal immunity is more effective in preventing and controlling the occurrence of avian influenza. The development of safe and effective mucosal adjuvants to induce an effective mucosal immune response and protection through mucosal vaccination is one of the future directions to achieve safer, more effective, and more convenient vaccinations [94].

For ruminants, although direct research is limited, the long-acting sustained-release mechanism of hydrogels can reduce the stress of frequent vaccinations in dairy cows [79], and the pH-responsive properties offer potential for developing rumen-stable oral vaccine delivery systems.

**Table 2:** Current research progress on hydrogel-based veterinary vaccines.

Hydrogel Category	Representative Materials	Advantages	Limitations	Suitable Vaccine Types	References
Coccidiosis vaccine	*Eimeria* spp.	Carrageenan/alginate composite hydrogel	Oral (microencapsulation)	2.3–2.5 mm gel beads protect oocysts, enhancing stability during storage	[85]
Rabies vaccine	Rabies virus	Metal-phenolic network (MPN) hydrogel	Subcutaneous injection	Single-dose immunization achieves 4.3× higher neutralizing antibodies	[27,28]
Newcastle disease vaccine	Newcastle disease virus (NDV)	Modified starch hydrogel	Liquid formulation	Maintains viral titer stability (<0.5 log10 EID50 loss) at 37 °C	[67]
H7N9 avian influenza vaccine	H7N9 influenza virus	Tetrapeptide hydrogel	Intramuscular injection	Increases micro-neutralization and hemagglutination inhibition (HI) titers in mice	[52]
Porcine epidemic diarrhea (PED)	PED virus	Alginate-chitosan hydrogel	Oral administration	Induces sustained mucosal IgA/IgG responses in mice (200%↑ vs. free antigen)	[80]
PRRSV vaccine	Porcine reproductive and respiratory syndrome virus	Thermosensitive hydrogel	Intranasal spray	Forms “mucosal mask” to prolong antigen retention in nasal epithelium	[77]
Canine rabies vaccine	Rabies virus	Thermo-responsive chitosan hydrogel	Subcutaneous injection	Generates 327.40 IU/mL neutralizing antibodies (200×↑ vs. liquid vaccine)	[87]
Feline coronavirus vaccine	Feline coronavirus	mRNA-LNP hydrogel composite	Under investigation	Demonstrates rapid antigen expression capability	[86]
Universal vaccine platform	Multi-pathogen	Reversible PEG-based hydrogel	Injectable depot	Stabilizes biologics at 65 °C via dynamic boronate crosslinking	[65]
DNA hydrogel vaccine	Toxin antigens	CpG-embedded DNA hydrogel	Subcutaneous injection	Enhances humoral/cellular immunity through TLR9 activation	[69]

Hydrogel delivery systems have significantly improved farm animal vaccination. Traditional vaccine injections often cause pain and stress, whereas hydrogel-based vaccines use needle-free methods such as microneedle patches and nasal sprays, effectively reducing stress responses and enhancing animal welfare [95,96]. These innovative delivery methods are easy to operate, reducing the labor-intensive requirements of traditional injections, saving time and resources. Additionally, needle-free administration avoids the risk of disease transmission through needles, improving biosafety.

### 4.3. Upgrade to Companion Animal Vaccines

The innovation of companion animal vaccines is gradually moving from traditional inactivated or attenuated vaccines to efficient, safe, and convenient next-generation technologies [97]. With the increase in pet medical needs and increasing awareness of the prevention and control of zoonotic diseases, vaccine research and development are focused on extending the protection cycle, improving immune targeting and simplifying the vaccination process.

The combination of novel delivery systems (such as hydrogel sustained-release platforms and microneedle transdermal patches) and molecular biology techniques (such as mRNA vaccines and recombinant protein antigens) is a solution to the lack of immunogenicity of traditional vaccines and the need for multiple booster vaccinations. The problem provides a new path [95]. For example, studies have shown that an inactivated rabies virus vaccine using a heat-responsive chitosan hydrogel as a carrier can induce high levels of persistent rabies virus neutralizing antibody (rVNA) in mice, and the concentration reaches an average of 327.40 IU/mL, which is more than 200 times greater than that of a liquid vaccine alone [98]. mRNA-LNP (lipid nanoparticle) technology has demonstrated the advantages of a rapid response and efficient expression in feline coronavirus vaccines.

Regarding delivery systems, mucosal immune hydrogels, such as nasal sprays and oral capsules, augment the protection of the respiratory and intestinal tracts by stimulating local immune responses. Concurrently, responsive materials, including pH-sensitive hydrogels, are capable of targeting lymph nodes to safeguard the respiratory and intestinal tracts by activating local immune responses and improving the efficiency of antigen presentation. The dissolvable hydrogel microneedle patch has been validated in a mouse model and may be used in pets in the future. This MN patch can achieve painless transdermal vaccination and long-term protection [99].

At the same time, breakthroughs in combination vaccines and therapeutic vaccines have significantly expanded application scenarios: multiple vaccines (such as combinations of canine quadruple vaccines and anthelmintic agents) simplify pet health management, whereas hydrogels loaded with tumor antigens and immune adjuvant platforms are constantly evolving [100,101]. For example, hydrogels can be combined with metal ions such as Mn^2+^ to form a porous structure, thereby achieving the efficient loading and release of immunomodulators. This design not only activates dendritic cells but also greatly enhances the infiltration of effector T cells, thus effectively inhibiting tumor growth [102]. Moreover, hydrogels can also serve as a unique platform to provide efficient antitumor therapy against the immunosuppressive tumor microenvironment [103]. Using these innovative hydrogel platforms, researchers can better control the delivery of tumor antigens and immune adjuvants. This combination therapy also provides new ideas for the immunotherapy of pet tumors (such as canine lymphoma and feline breast cancer).

The customized design of a hydrogel vaccine delivery system can effectively address interindividual differences in companion animals and provide more accurate and efficient immune protection. This technological advancement provides new directions and possibilities for the future development of vaccine delivery systems.

## 5. Conclusions

Hydrogel technology has enormous application potential in the field of veterinary vaccines. In laboratory studies, it has demonstrated many advantages, such as good biocompatibility, controlled release and sustained release ability, and immune enhancement properties [104]. Studies on various delivery methods, such as transdermal, oral, and implantable sustained release, have proven the feasibility of these methods in different application scenarios. However, some challenges still need to be overcome to achieve translation from the laboratory to pasture.

In terms of safety, further evaluation is needed to assess its long-term impact. The hydrogel vaccine delivery system has shown good biocompatibility overall in experimental models, but there are still some potential side effects that need to be given attention. For example, certain hydrogel systems may cause unwanted inflammatory responses in the body or exhibit insufficient biodegradability over long-term use. For instance, PNP hydrogels (loaded with mRNA/LNP) initially recruit neutrophils and monocytes upon injection [105], but if the adjuvant dose (such as 3M-052) is too high, it may prolong the inflammatory period. Synthetic hydrogels (like PEI-modified GO) may release low-molecular-weight polyethyleneimine (LPEI) during degradation, which can cause cell membrane damage at high concentrations. Additionally, while nano-engineered polymer hydrogel capsules show significant immunostimulatory capabilities in the body, their long-term impact on the immune system still requires further assessment [106].

Economically, the development and application of hydrogel vaccine delivery systems could reduce vaccination costs. Traditional vaccinations require professional medical personnel and equipment, whereas hydrogel systems can be delivered via innovative methods such as microneedles, patches, and inhalable systems, which are relatively simpler and more cost-effective [8]. Moreover, hydrogels can provide sustained antigen release within the body, a feature that extends the duration of the immune response, thereby reducing the frequency and dosage of vaccinations.

Overall, hydrogel vaccine delivery systems offer significant advantages in enhancing vaccine efficacy and safety, and they may also have a positive economic impact on vaccination efforts by reducing costs and simplifying the administration process.

By strengthening basic research, we can gain an in-depth understanding of the interaction mechanism between hydrogels and the animal immune system to provide a more solid theoretical basis for their application. Moreover, more clinical trials should be carried out to verify their efficacy and safety in actual breeding environments, establish a complete quality control and supervision system, and promote the extensive application of hydrogel technology in the field of animal immunization, from the laboratory to clinical practice. These results can be transformed into actual application on the ranch.

## Figures and Tables

**Table 1:** Types and substances for hydrogel-based vaccines.

Hydrogel Category	Representative Materials	Advantages	Limitations	Suitable Vaccine Types	References
Natural hydrogels	Alginate, chitosan, hyaluronic acid	High biocompatibility, biodegradability, low toxicity	Low mechanical strength, swelling instability	Oral/mucosal vaccines (e.g., PEDV)	[13,18,19]
Synthetic hydrogels	PEG, Poloxamer 407/188	High stability, controlled release, easy functionalization	Low bioactivity, potential degradation byproducts	Long-acting injectables (e.g., rabies)	[14,20]
Composite hydrogels	Nanoparticle-hydrogel (e.g., GO/PEI)	Targeted delivery, synergistic immune activation	Complex fabrication, safety validation needed	mRNA vaccines/cancer immunotherapy	[21,22]
Stimuli-responsive hydrogels	Thermosensitive chitosan, pH-sensitive phosphorylated chitosan	Spatiotemporal-controlled release, enhanced mucosal retention	Environmental sensitivity affects performance	Intranasal influenza vaccines, gut-targeted oral vaccines	[11,12]

## Data Availability

No new data were created or analyzed in this study.
